# Age-specific determinants of psychiatric outcomes after the first COVID-19 wave: baseline findings from a Canadian online cohort study

**DOI:** 10.1186/s13034-023-00560-8

**Published:** 2023-02-06

**Authors:** S. Evelyn Stewart, John Best, Robert Selles, Zainab Naqqash, Boyee Lin, Cynthia Lu, Antony Au, Gaelen Snell, Clara Westwell-Roper, Tanisha Vallani, Elise Ewing, Kashish Dogra, Quynh Doan, Hasina Samji

**Affiliations:** 1 Department of Psychiatry, University of British Columbia, Vancouver, BC Canada; 2 British Columbia Children’s Hospital Research Institute, Vancouver, BC Canada; 3 Faculty of Health Sciences, Simon Fraser University, Burnaby, BC Canada; 4 Faculty of Medicine, University of British Columbia, Vancouver, BC Canada; 5 Department of Pediatrics, University of British Columbia, Vancouver, BC Canada; 6 British Columbia Centre for Disease Control, Vancouver, BC Canada

**Keywords:** COVID, Outcome, Age, Psychiatry, Access, Determinant, Child, Adolescent, Poverty, LGBTQ, Gender

## Abstract

**Background:**

Canadians endured unprecedented mental health (MH) and support access challenges during the first COVID-19 wave. Identifying groups of individuals who remain at risk beyond the acute pandemic phase is key to guiding systemic intervention efforts and policy. We hypothesized that determinants of three complementary, clinically actionable psychiatric outcomes would differ across Canadian age groups.

**Methods:**

The Personal Impacts of COVID-19 Survey (PICS) was iteratively developed with stakeholder feedback, incorporating validated, age-appropriate measures. Baseline, cross-sectional online data collected between November 2020-July 2021 was used in analyses. Age group-specific determinants were sought for three key baseline MH outcomes: (1) current probable depression, generalized anxiety disorder, obsessive–compulsive disorder and/or suicide attempt during COVID-19, (2) increased severity of any lifetime psychiatric diagnosis, and (3) inadequate MH support access during COVID-19. Multivariable logistic regression models were constructed for children, youth (self- and parent-report), young adults (19–29 years) and adults over 29 years, using survey type as a covariate. Statistical significance was defined by 95% confidence interval excluding an odds ratio of one.

**Results:**

Data from 3140 baseline surveys were analyzed. Late adolescence and early adulthood were identified as life phases with the worst MH outcomes. Poverty, limited education, home maker/caregiver roles, female and non-binary gender, LGBTQ2S + status and special educational, psychiatric and medical conditions were differentially identified as determinants across age groups.

**Interpretation:**

Negative psychiatric impacts of COVID-19 on Canadians that include poor access to MH support clearly persisted beyond the first wave, widening pre-existing inequity gaps. This should guide policy makers and clinicians in current and future prioritization efforts.

**Supplementary Information:**

The online version contains supplementary material available at 10.1186/s13034-023-00560-8.

## Introduction

### Pre-Pandemic mental health (mh) and support access across the lifespan

One in five Canadians are affected by mental illness [64], with difficulties typically emerging during adolescence and early adulthood [65]. Across age groups, depression and generalized anxiety are particularly common [21, 42]. Vulnerability to mental illness is influenced by a combination of genetic risk factors and socio-environmental determinants including, for Canadian children and youth, low family/neighborhood income, limited education/employment opportunities, housing/food insecurity, and early life stressors [65]; and for Canadian adults, low socioeconomic status and educational attainment, unemployment, female and non-binary gender, lesbian/gay/bisexual orientation, and chronic physical disease/disability, among others [3, 49, 66, 68, 69].

Fortunately, the long-term morbidity of most psychiatric illnesses can be mitigated through early intervention [8]*.* In fact, access to MH service mediates relationships between determinants and outcomes among young Canadians [65]. However, many Canadians have difficulty accessing MH support and those that cannot may be among the most vulnerable. As per the 2012 Canadian Community Health Survey, 20% accessed formal or informal MH support [60] and 12% reported unmet MH needs. Those with unmet needs were younger, more commonly had substance use disorders, and had fewer interactions with professionals [14, 57].

### Initial pandemic impacts on MH

At this point, it is well-established that the initial wave of COVID-19 was associated with global impacts on mental health, especially anxiety and depression [10]. Our systematic review of MH data from the first COVID-19 wave identified increased depression and anxiety symptoms among children and youth, with older adolescents, females, neurodiverse and chronically ill individuals appearing most underserved [51]. Increased anxiety and depression symptoms also emerged among adults but were reportedly restricted to the early months of 2020 [2, 11]. Obsessive–compulsive disorder (OCD) symptoms increased during the pandemic, regardless of diagnostic status [26, 34]. Globally, risk appeared greatest in younger versus older adults [63], in the LGBTQ + community [45] and among adults with pre-existing psychiatric diagnoses [27]. However, Canada-specific data indicated divergent influences of pre-existing psychiatric diagnosis with deterioration in some and improvement in others [9].

### Ongoing pandemic impacts on MH

Beyond the initial impact of the pandemic, Canadians have endured unprecedented impacts to daily activities across multiple COVID-19 waves. Given that the ‘incubation period’ for psychiatric problems typically exceeds that for infectious illness, it is critical to build upon literature from the first wave and identify the extent to which clinically significant psychiatric problems emerged through the pandemic, what social determinants are most predictive of these concerns, and what needs for MH support remain unmet. Further, as life experience and developmental life stage may differentially influence vulnerability and resilience [61], identification of age group-specific determinants provides an important contribution to guide policy and intervention.

This study responds to calls for examination of long-term pandemic MH impacts and service provision in different age groups [20, 28, 40, 51]. Broad, transdiagnostic MH outcomes were intentionally defined based upon their actionable and societal relevance, including expectedly common or potentially lethal psychiatric problems, increased severity of any clinically-diagnosed psychiatric condition and inadequate MH support access. GAD, depression and OCD were selected for focus based on first wave studies [10, 34, 51, 63]. Further, since increased anxiety, sadness and contamination avoidance during COVID-19 would be expected and not necessarily indicative of a need for clinical attention among Canadians, we also required the presence of symptom-related impairment, using a more conservative approach than many early pandemic studies [20]. Suicide attempts during COVID-19 were also included, given pandemic associated risk [6], interconnections with psychiatric illness [38, 70] and associated mortality risk [17].

We hypothesized that distinct age group-specific determinants would predict: (1) common or high lethality psychiatric problems [defined by at least one of: suicide attempt during COVID-19, or probable Generalized Anxiety Disorder (GAD), Major Depressive Disorder (MDD) or Obsessive–Compulsive Disorder (OCD) at baseline]; (2) increased severity of any diagnosed lifetime psychiatric illness during COVID-19; and (3) unmet MH support needs.

## Method

### Study design

The online survey study protocol was approved by the UBC Children’s and Women’s Research Ethics Board (see Additional file 5). Baseline data collection transpired from November 2020- July 2021 after the first COVID-19 wave in Canada (see Additional file 2). Data from follow-up surveys are not presented in this paper. See details in the Checklist for Reporting Results of Internet E-Surveys (CHERRIES) [12] (see Additional file 1).

### Selection and description of participants

Multi-source recruitment via social media and targeted, non-random approaches [43] aimed to recruit a representative sample of 3000 Canadians for maximal result generalizability (Table 1). Canadians over 8 years (years) were eligible. Non-Canadians, suspected fraudulent respondents, those without stated age and non-completers were excluded (see Additional file 5).

**Table 1:** Participant characteristics by age group and informant

	Child by parent-report (< 8 year)	Youth by parent-report (8–18 year)	Youth self-report (8–18 year)	Young adult self-report (19–29 year)	Adult self-report (30 + year)	Overall Sample	Canadian population reference^1^
	N = 192	N = 289	N = 137	N = 586	N = 1.936	N = 3.140	
Gender							
Female	90 (47%)	136 (47%)	78 (57%)	446 (76%)	1571 (81%)	2321 (74%)	50.9%
Male	98 (51%)	142 (49%)	55 (40%)	114 (19%)	324 (17%)	733 (23%)	49.1%
Non-binary	4 (2.1%)	11 (3.8%)	4 (2.9%)	26 (4.4%)	41 (2.1%)	86 (2.7%)	
LGBTQ2S +							
Yes	NA	NA	16 (12%)	172 (29%)	197 (10%)	NA	6.4%; 3.1%^2^
No	NA	NA	113 (82%)	391 (67%)	1697 (88%)	NA	93–97%
Prefer not to answer	NA	NA	8 (5.8%)	23 (3.9%)	42 (2.2%)	NA	NA
Ethnicity							
Metis/1st Nations	9 (4.7%)	7 (2.4%)	4 (2.9%)	8 (1.4%)	40 (2.1%)	68 (2.1%)	6.2%
East/central Asian	9 (4.7%)	12 (4.2%)	3 (2.2%)	47 (8.0%)	85 (4.4%)	156 (5.0%)	9.2%
South Asian	28 (15%)	38 (13%)	6 (4.4%)	42 (7.2%)	56 (2.9%)	170 (5.4%)	5.7%
White	142 (74%)	220 (76%)	94 (69%)	383 (65%)	1,539 (79%)	2378 (76%)	78%^3^
Multi	3 (1.6%)	3 (1.0%)	21 (15%)	70 (12%)	110 (5.7%)	207 (6.6%)	NA
Other	1 (0.5%)	9 (3.1%)	9 (6.6%)	36 (6.1%)	106 (5.5%)	161 (5.1%)	NA
Immigrant	9 (4.7%)	19 (6.6%)	8 (5.8%)	92 (16%)	346 (18%)	474 (15%)	8.3%; 21.9%^4^
Parent of 0–18 years	NA	NA	NA	43 (7.3%)	1,067 (55%)	NA	37.5%
Child mean y (SD)	4.4 (2.3)	13.2 (3.0)	12.7 (2.9)	NA	NA	NA	NA
Child with special needs	18 (9.4%)	87 (30%)	NA	NA	NA	NA	10.9%; 4.6%^5^
Rural setting	13 (6.9%)	15 (5.5%)	9 (6.9%)	32 (6.1%)	158 (8.5%)	227 (7.2%)	16.1%
Northern setting	6 (3.2%)	7 (2.6%)	1 (0.8%)	7 (1.4%)	40 (2.2%)	61 (1.9%)	5.3%
Education (parent/adult)							
< Bachelor’s	46 (24.3%)	98 (35.4%)	43 (32%)	287 (49.3%)	728 (38.1%)	1202 (38%)	71.5%
Bachelor’s degree	70 (36%)	93 (33%)	47 (35%)	244 (42%)	635 (33%)	1089 (35%)	19%
Graduate degree	73 (38%)	86 (31%)	45 (33%)	51 (8.7%)	550 (28%)	805 (26%)	9.5%
Prefer not to answer	3 (1.6%)	3 (1.1%)	0 (0%)	4 (0.7%)	23 (1.2%)	33 (1.0%)	NA
(Missing)	0	9	2	0	0	11	NA
Annual household income							
< $75 000	19 (16%)	25 (14%)	12 (16%)	154 (63%)	275 (25%)	485 (29%)	53%
$75 000 to 99 999	11 (10%)	26 (15%)	12 (16%)	17 (6.9%)	176 (16%)	242 (14%)	14.5%
$100 000 to 149 999	47 (43%)	35 (20%)	16 (22%)	34 (14%)	282 (26%)	414 (24%)	17.6%
> $149 999	30 (27%)	73 (41%)	30 (41%)	9 (3.7%)	290 (26%)	432 (25%)	14.7%
Prefer not to answer	3 (2.7%)	17 (9.7%)	4 (5.4%)	32 (13%)	72 (6.6%)	128 (7.5%)	NA
(Missing)	82	113	63	340	841	1439	NA
Food Insecure	3 (2.5%)	26 (9.0%)	13 (9.5%)	71 (12%)	212 (12%)	325 (11%)	14.6%^**6**^
(Missing)	0	0	0	7	136	143	
Number in home mean (SD)	3.00 (1.14)	3.26 (1.15)	3 (1)	2.24 (1.42)	2.32 (1.50)	2.48 (1.47)	2.4
Lifetime psychiatric d/o	10 (5.2%)	79 (27%)	30 (22%)	286 (49%)	659 (34%)	1064 (34%)	33.1%^**7**^
Alcohol/substance use problem	0 (0%)	2 (0.7%)	1 (0.7%)	17 (2.9%)	59 (3.0%)	79 (2.5%)	12%; 2%^8^
Medical condition	27 (14%)	53 (18%)	30 (22%)	253 (43%)	905 (47%)	1268 (40%)	22%; 44%^9^
Assessment period							
Pre March 2021	88 (46%)	164 (57%)	74 (54%)	264 (45%)	1064 (55%)	1654 (53%)	NA
Post Feb 2021	104 (54%)	125 (43%)	63 (46%)	322 (55%)	872 (45%)	1486 (47%)	NA

Numbers (and percentages) are presented unless noted otherwise

^1^Statistics Canada. 2017, unless noted. See Additional file 3 for further details re: characteristics and references for Canadian statistics

^2^%Lesbian/Gay/Bisexual (2015–2018) 15–24 year= 6.4%; 25–64 year = 3.1%

^3^% Indicating not being a visible minority

^4^% Immigrants among < 15 year = 8.3%; 15 year +  = 21.9%

^5^ < 15 year = 10.9%; 15–19 year = 4.6%

^6^Includes psychiatric and substance disorders

^7^15–25 y = 12%; 45–65 year = 2%

^8^0–18 y = 22%; > 19 year

### Primary measurements/outcomes

The Personal Impacts of COVID-19 Survey (PICS) combines validated questionnaires and novel items, iteratively co-created with stakeholders between April and November 2020 (see acknowledgment section). Versions included self-reports for youth and adults, and parent-reports for children/youth. Items characterized individuals, pandemic impacts and pandemic-era supports.

#### Outcome 1: Probable GAD, depression, OCD and/or pandemic-era suicide attempt

GAD, depression and OCD symptoms were identified via validated measures: the Generalized Anxiety Disorder Scale (GAD-7) [36, 55], Patient Health Questionnaire for Depression (PHQ-9) [29, 46] and the Obsessive–Compulsive Inventory–Revised (OCI-R) and -Child Version (OCI-CV) [15, 16], with clinical threshold scores of 10, 10, 21 and 11, respectively. “*How difficult have these problems made it for you to do your work, take care of things at home, or get along with other people?”* queried impact required for GAD and depression. Item 2 from adult/child versions of the Yale-Brown Obsessive Compulsive Scale [24, 52] queried impact required for OCD. Above-threshold scores with concurrent impairment, or a reported COVID-19-era suicide attempt provided a dichotomous indicator of clinically relevant psychiatric problems.

#### Outcome 2: Increased severity of any diagnosed psychiatric illness

Those reporting one or more lifetime psychiatric diagnoses by a health professional were asked for each disorder whether its severity changed during COVID-19, using a 5-point Likert scale with item scoring from “much worse” to “much better”.

#### Outcome 3: unmet MH support needs

Unmet MH support needs were captured by asking respondents if they had needed but not received support for their MH, with response choices of “yes” or “no”.

### Statistical analyses

Data processing and statistical models were constructed using R version 4.1.1 (r-project.org). Data were initially summarized using descriptive statistics, including mean scores and standard deviations for continuous measures and numbers and percentages for categorical variables. Data were identified as missing at the item-level. Forty missing item-level data sets were imputed using *‘mice’* (version 3.13.0) [62]. Imputed values’ quality was assured by evaluating imputed values distributions and trace plots for proper mixing and spike absence in the iterations. Subsequent analyses were conducted on each imputed data set with estimates pooled using Rubin’s rule [48] and degrees of freedom calculated using Barnard-Rubin adjustment [5].

Primary analyses involved multivariable logistic regression models, in which the binary outcome variable was regressed on primary variables of interest and covariates. Separate regression models were constructed for each age group (parent-report on children under 8 years, parent-report on youth 8–18 years, youth self-report 8–18 years, and self-reports on young adults 19–29 years, and adults over 29 years). Due to survey differences, not all included variables were identical across models. Key variables in all models included: age, gender, race (white versus other), residence (rural versus urban), poverty (income under $75,000 and/or food insecurity), lifetime psychiatric diagnosis, medical condition and assessment period (dichotomized starting March 1, 2021). Additional variables of interest were special education needs of 0–18 year old (all models except adult self-report); LGBTQ2S + status (all models except parent-report); and current employment status (adult self-report models). For adult models, survey type was included as a covariate. Model results are presented using odds ratios (OR) and 95% confidence intervals. Statistical significance was identified based on 95% confidence intervals that do not include the OR of one (equivalent to a nominal p value < 0.05).

## Results

### Baseline participants

Data from 3140 participant baseline surveys were included for analyses (see Additional file 3). Table 1 characterizes demographic and traditional MH determinant prevalence across age groups relative to the Canadian population (indicating degree of anticipated study result generalizability). Additional details are provided in Additional file 4. Across age groups, individuals identifying as female, non-binary, non-white, LGBTQ2S + , immigrants, urban residents, and those with medical conditions were adequately represented, as were children and youth with special educational needs. Under-represented characteristics across age groups included specific ethnicities (Metis/First Nations, east/central Asians), rural/northern residents, low educational attainment, living in poverty (except in young adults) and, among adults, male gender.

#### Determinants of current GAD, depression or OCD and/or pandemic-era suicide attempt

**Fig. 1:**
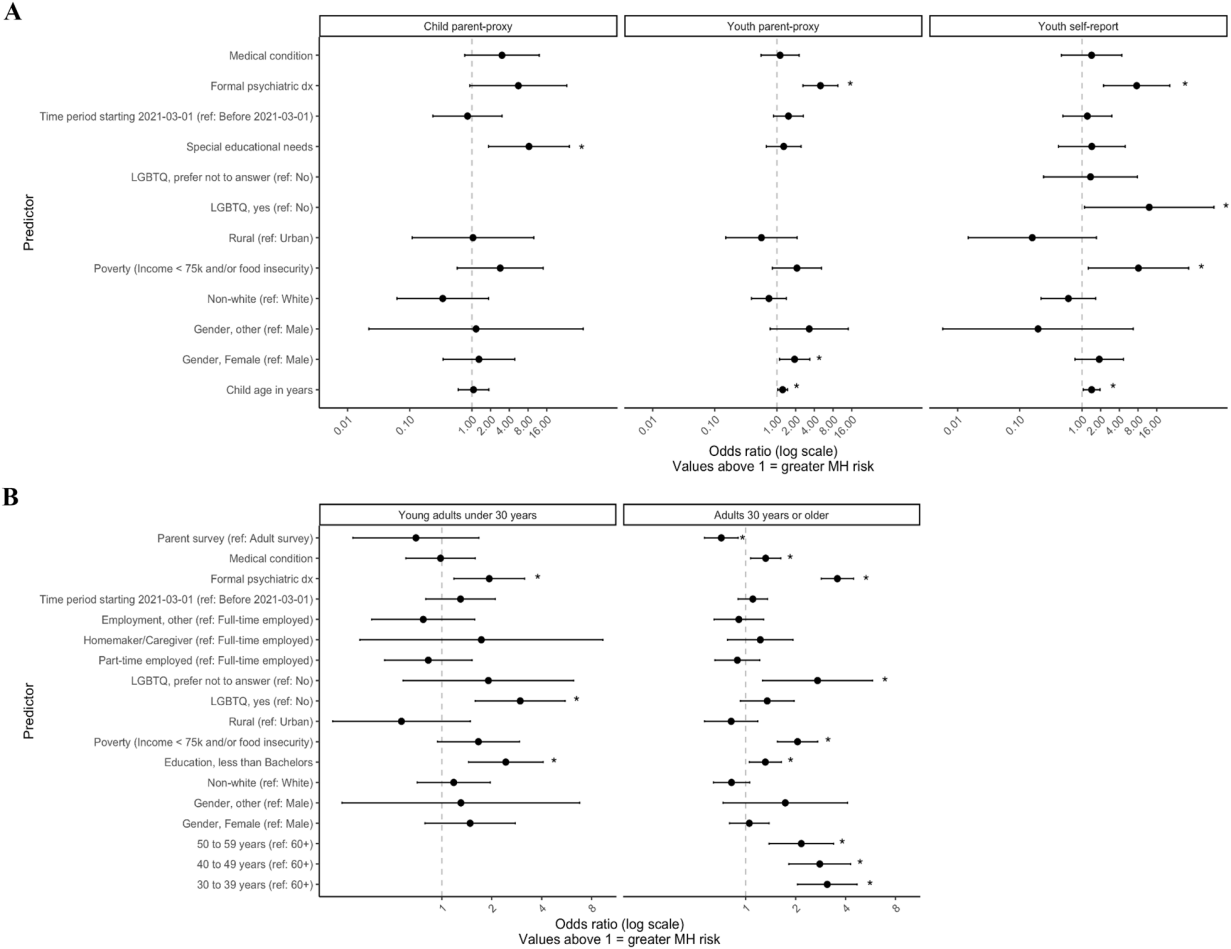
Predictors of selected psychiatric problems (any of suicide attempt during COVID-19 or GAD/depression/OCD with impairment) by age groups. *Note.* * *p* < .05. Panel A: children (0–7 year) by parent-report and youth (8–18 year) by parent- and self-reports Panel B: young adults (19–29 year) and adults over 30 year by self-report

Prevalence of components of outcome #1 as reported at baseline across age groups are detailed in Table 2. Associated factors differed across age groups (Fig. 1).

**Table 2:** Age-specific rates of current GAD, depression, OCD and pandemic-era suicide attempt^1^

	Child < 8 year (parent-report) N = 192	Youth 8–18 year (parent-report) N = 289	Youth 8–18 year (self-report) N = 137	Young adult 19–29 y (self-report) N = 586	Adult 30 + year (self-report) N = 1936
Probable GAD	10 (5.2%)	69 (24%)	42 (31%)	319 (55%)	684 (38%)
Probable Depression	8 (4.2%)	76 (26%)	48 (35%)	325 (56%)	839 (46%)
Probable OCD	9 (4.7%)	52 (18%)	44 (33%)	136 (24%)	210 (12%)
Suicide attempt	1 (0.5%)	18 (6.2%)	6 (4.4%)	26 (4.5%)	25 (1.4%)
Any of the above^1^	**16 (8.3%)**	**116 (40%)**	**63 (47%)**	**390 (68%)**	**981 (55%)**
Missing	0	0	2	13	158

Bold values indicate the age-sepcific numbers for primary outcome 1

^1^For Canadian reference rates, see Additional file 3 Appendix Table S2

^2^Representing outcome #1 used in risk prediction analyses

Figure 1A presents results of multivariable logistic regression models pertaining to selected psychiatric problems (with binary dependent variable indicating the presence of one or more of probable GAD, depression, OCD or suicide attempt) in children (0–7 years) and youth (8–18 years). Figure 1B presents associated factors among young adults (19–29 years) and adults over 29 years.

#### Determinants of Worsening Severity of Any Lifetime Psychiatric Diagnoses during COVID-19

Data on lifetime psychiatric diagnoses, rates of increased severity since the onset of COVID-19, and attribution of increased severity to COVID-19 is provided in Table 3.

**Table 3:** Lifetime psychiatric diagnoses, increased severity and attribution to COVID-19

	Child by parent-report (< 8 year)	Youth by parent-report (8–18 year)	Youth self-report (8–18 year)	Young adult self-report (19–29 year)	Other adult self-report (30 + year)
	N = 192	N = 289	N = 137	N = 586	N = 1,936
GAD					
Lifetime diagnosis	6 (3.1%)	43 (15%)	16 (12%)	193 (33%)	339 (18%)
Increased severity	5 (83%)	33 (77%)	14 (88%)	155 (80%)	241 (71%)
Increase due to COVID-19	5 (100%)	26 (79%)	7 (50%)	119 (77%)	192 (80%)
Depression					
Lifetime diagnosis	0 (0%)	28 (9.7%)	12 (8.8%)	200 (34%)	496 (26%)
Increased severity	NA	18 (64%)	11 (92%)	143 (72%)	331 (67%)
Increase due to COVID-19	NA	16 (89%)	7 (64%)	109 (76%)	259 (78%)
OCD					
Lifetime diagnosis	1 (0.5%)	20 (6.9%)	10 (7.3%)	43 (7.3%)	47 (2.4%)
Increased severity	0 (0%)	15 (75%)	5 (50%)	29 (67%)	24 (51%)
Increase due to COVID-19	NA	12 (80%)	3 (60%)	23 (79%)	14 (58%)
Other psychiatric disorder^1^					
Lifetime diagnosis	6 (3.1%)	48 (17%)	19 (14%)	189 (32%)	383 (20%)
Increased severity	3 (75%)	21 (48%)	16 (84%)	103 (58%)	192 (54%)
Increase due to COVID-19	3 (100%)	13 (62%)	13 (81%)	75 (73%)	138 (72%)
Any psychiatric disorder^2^					
Any lifetime diagnosis	10 (5.2%)^3^	79 (27%)^3^	30 (22%)^**3**^	286 (49%)^4^	659 (34%)^4^
Increase severity of any	5 (50%)	49 (62%)	19 (63%)	212 (74%)	450 (68%)
Increase in any due to COVID-19	5 (100%)	37 (76%)	11 (58%)	173 (82%)	364 (81%)

*Note.* “Worsening due to COVID-19” is only asked of those reporting increased severity

^1^Comprising other anxiety, PTSD, ADHD, tic, psychosis, and bipolar disorders

^2^Comprising all disorders above

^3^Ontario Child Health Survey reference for GAD, depression, any psychiatric disorder, respectively: 4–11 y: 3.4%, 1.1%, 18.1%; 12–17 y: parent-report 5.5%, 5.2%, 18.2%; self-report 9.7%, 7.3%, 21.8%

^4^Statistics Canada reference for GAD, depression, any psychiatric disorder, respectively: 15–24 y: 2.4%, 7.1%, 18.5%; 25–64 y: 3.0%, 5.0%, 9.8%

**Fig. 2:**
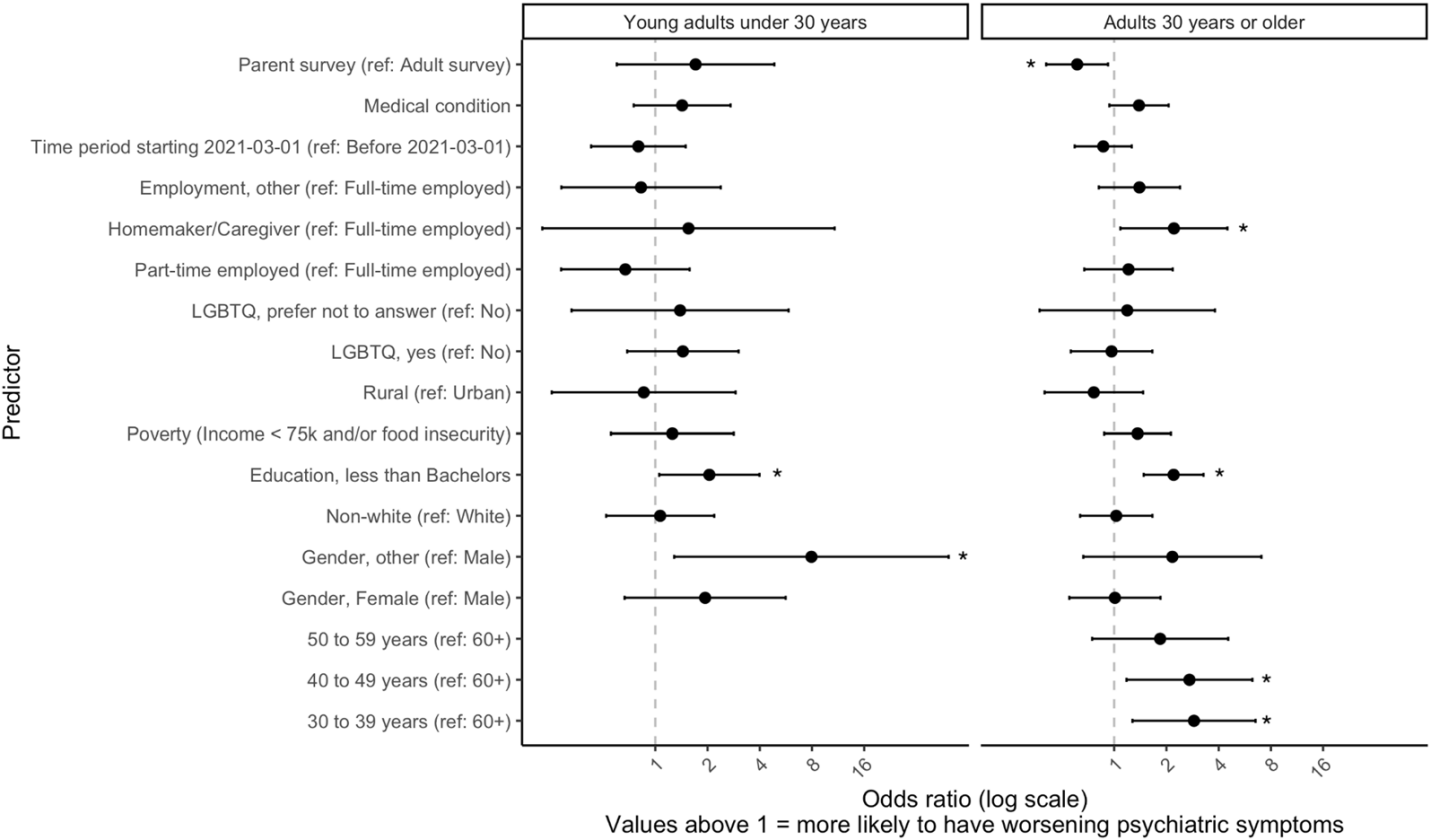
Predictors of increased severity for any lifetime psychiatric diagnosis among adults during COVID-19**.** *Note.* * *p* < .05**.** *Mental Health Support: Means Received, Satisfaction and Predictors of Unmet Needs*

Regression models for increased diagnostic severity were not constructed for children or youth due to limited sample sizes of those with lifetime diagnoses. Figure 2 shows regression results for the young adult and adults over 30 years age groups.

Table 4 provides descriptive data on pandemic-era MH support across age groups as reported following the first wave.

**Table 4:** Mental health support during and following the first wave of COVID-19

Variable	Child by parent-report (< 8 year)	Youth by parent-report (8–18 year)	Young adult self-report (19–29 year)	Adult over 30 year self-report
	N = 192	N = 289	N = 586	N = 1,936
Mental health support since start of pandemic				
No	95 (49%)	138 (48%)	217 (39%)	844 (50%)
Yes	18 (9.4%)	100 (35%)	288 (52%)	657 (39%)
No support needed	78 (41%)	47 (16%)	45 (8.1%)	189 (11%)
Prefer not to answer	1 (0.5%)	4 (1.4%)	3 (0.5%)	8 (0.5%)
(Missing)	0	0	33	238
Satisfaction with care since start of pandemic				
Very unsatisfied	2 (15%)	8 (12%)	17 (6.9%)	47 (8.6%)
Slightly unsatisfied	1 (7.7%)	12 (18%)	32 (13%)	58 (11%)
Neutral	1 (7.7%)	7 (10%)	68 (27%)	109 (20%)
Slightly satisfied	3 (23%)	16 (24%)	60 (24%)	117 (21%)
Very satisfied	6 (46%)	25 (37%)	69 (28%)	205 (37%)
Prefer not to answer	0 (0%)	0 (0%)	2 (0.8%)	13 (2.4%)
Not applicable	179	221	338	1387
Type of support				
Email	13 (6.8%)	70 (24%)	104 (18%)	126 (6.5%)
In person	12 (6.2%)	59 (20%)	213 (36%)	354 (18%)
Online or virtual	5 (2.6%)	27 (9.3%)	108 (18%)	319 (16%)
Telephone	0 (0%)	5 (1.7%)	16 (2.7%)	138 (7.1%)
Care needed but not received				
No	163 (85%)	203 (70%)	313 (57%)	1,191 (70%)
Yes	23 (12%)	79 (27%)	211 (38%)	436 (26%)
Prefer not to answer	6 (3.1%)	7 (2.4%)	29 (5.2%)	71 (4.2%)
(Missing)	0	0	33	238

(of note, these items were not included in youth self-report)

**Fig. 3:**
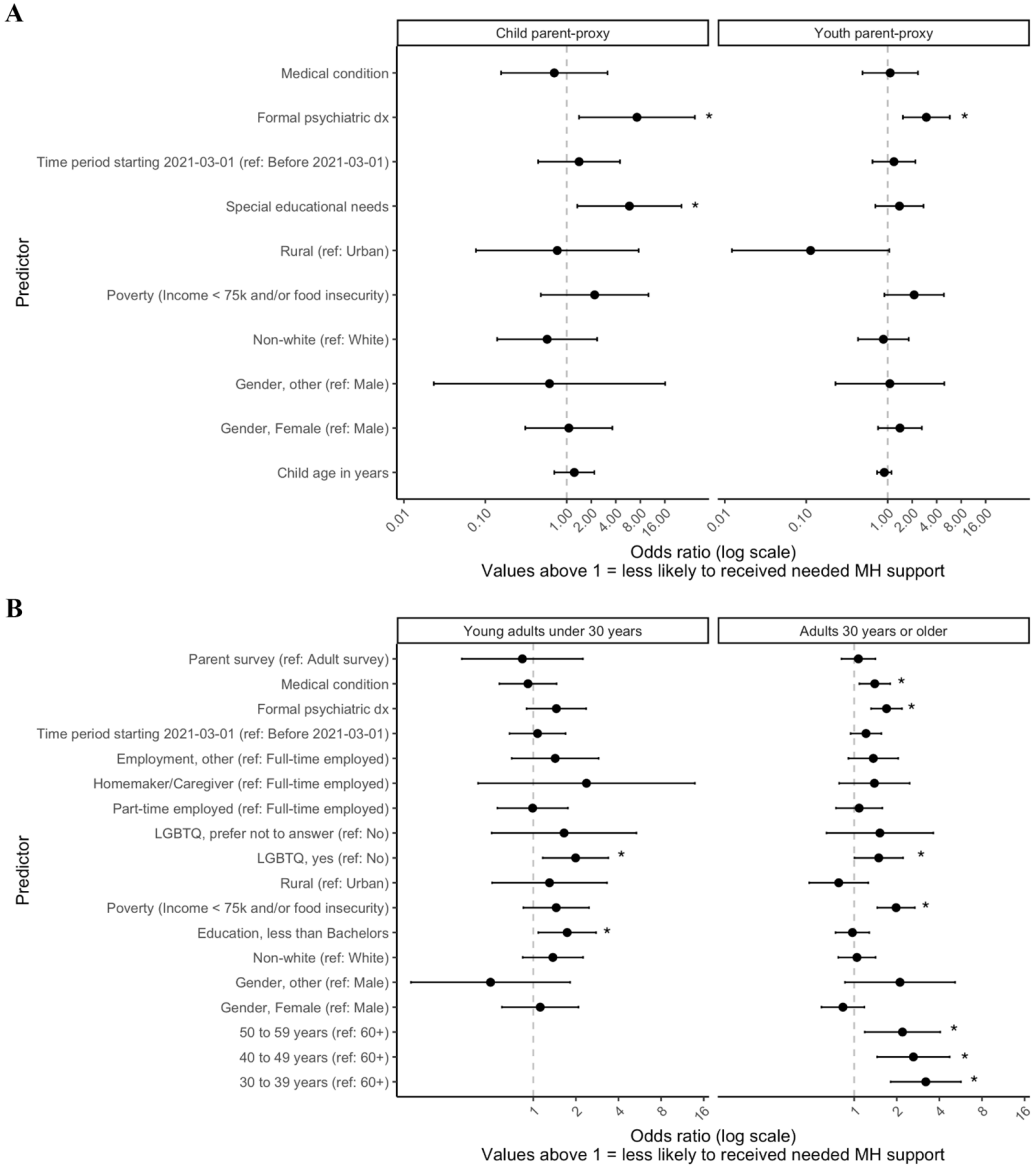
Determinants of unmet mental health support needs among children and youth (panel **A**) and adults (panel **B**). *Note.* * *p* < .05

Determinants of unmet MH support needs during the COVID-19-era are shown in Fig. 3a (for children and youth) and Fig. 3b (for young adults and adults 30 years and older.

## Discussion

### Key findings

This study adds to pre-pandemic and first wave evidence, with findings that negative MH impacts of COVID-19 persist, and that these impacts and their predictors differ across age groups (Table 5). While COVID-19 infection has not spared any sector of society, MH sequelae of the pandemic appear to have disproportionately impacted Canadians transitioning from childhood to adulthood. Moreover, pre-existing MH disparities have been amplified, with ongoing or increasing influence of determinants such as poverty, limited education, LGBTQ2S + status, female and non-binary gender, medical and psychiatric illness.

**Table 5:** Age-specific determinants of psychiatric outcomes after the first COVID-19 wave across ages

Determinant	Child-PR OR (95% CI)	Youth-PR OR (95% CI)	Youth-SR OR (95% CI)	Young Adult OR (95% CI)	Adult 30y + OR (95% CI)
Age					
Older from 8–18 year	NA	1.23 (1.03–1.48)^a^	1.43 (1.05–1.95)^a^	NA	NA
Younger (30–39 year) vs 60 + y	NA	NA	NA	NA	2.88 (1.27–6.52)^b^
					3.21 (1.81–5.68)^c^
					3.10 (2.05–4.69)^P^
Younger (40–49 year) vs 60 + y	NA	NA	NA	NA	2.62 (1.45–4.73)^c^
					2.79 (1.82–4.29)^a^
					2.71 (1.18–6.26)^b^
Younger (50–59 year) vs 60 + y	NA	NA	NA	NA	2.16 (1.38–3.39)^a^
					2.19 (1.19–4.05)^c^
Gender and orientation					
Female^1^	ns	1.92 (1.10–3.37)^a^	ns	ns	ns
Non-binary^1^	ns	ns	ns	7.94 (1.28–49.10)^b^	ns
LGBTQ2S +	ns	ns	12.1 (1.1–133)^a^	2.97 (1.59–5.54)^a^	1.49 (1.00–2.22)^c^
				1.99 (1.17–3.29)^c^	
Education, poverty, homemaker/caregiver and parental status					
Limited education	ns	ns	ns	2.43 (1.45–4.07)^a^	1.31 (1.05–1.64)^P^
				2.05 (1.05–3.98)^b^	2.20 (1.48–3.27)^S^
				1.74 (1.09–2.78)^c^	
Poverty	ns	ns	8.10 (1.26–51.90)^a^	ns	2.05 (1.55–2.71)^PP^
					1.98 (1.46–2.68)^c^
Homemaker/caregiver	NA	NA	NA	ns	2.21 (1.09–4.50)^S^
Non-parent of 0–18y	NA	NA	NA	ns	0.61 (0.41–0.92)^S^
Developmental conditions, psychiatric and medical diagnoses					
Special Education Needs	8.28 (1.86–37.00)^a^	ns	ns	NA	NA
	5.88 (1.35–25.60)^c^				
Lifetime psychiatric diagnosis	7.27 (1.42–37.4)^c^	5.01 (2.62–9.57)^aa^	7.59 (2.23–25.90)^aa^	1.93 (1.18–3.15)^a^	3.57 (2.85–4.46)^a^
		2.98 (1.54–5.80)^c^			1.69 (1.32–2.18)^c^
Medical condition	ns	ns	ns	ns	1.32 (1.07–1.62)^a^
					1.40 (1.09–1.80)^c^

*Ns* non-significant, *NA* not applicable

^a^select psychiatric problem (current depression, GAD, OCD or suicide attempt during COVID-19) as reported at baseline

^b^increased severity of any psychiatric diagnosis during COVID-19

^c^unmet MH support needs during COVID-19

^1^relative to male gender

#### Prevalence

Rates of GAD, depression, OCD and unmet MH support needs were multiple-fold of those reported by Canadian pre-pandemic studies including the 2014 Ontario Child Health Survey [21, 42] and the 2012 Canadian Community Health Survey [57] (see Additional file 3). As noted, feelings of sadness and worry and washing behaviors are all expected during an infectious pandemic, and as such, caution was required to not pathologize or label individuals based upon these singular symptoms. The use of validated psychiatric measures and the requirement for associated impairment add confidence to our conclusion that prevalence remained markedly amplified compared to pre-pandemic Canadian rates. Moreover, while Aknin et al. [2] reported on resolution of anxiety and depression symptoms beyond early 2020, our study found that these continued Aknin at least until mid-2021.

#### Age

The age-specific patterns parallel separate results of Canadian child [9, 51] and adult [11, 41] studies and those from other countries [10, 19]. Specifically, older adolescents and young adults report highest levels of common or high lethality psychiatric problems. Emergence of mental illness generally occurs between 14 and 25 years [54], representing a sensitive period of development into mature adulthood. As such, it follows that pandemic impacts on social isolation, educational environments, job loss and MH service availability disproportionately affected youth and young adults [59]. Amplifying challenges, this life phase also aligns with lapse of service between child and adult MH systems that exist in Canada and globally [39, 53].

#### Gender and orientation

Relative to males, female youth and non-binary young adult participants were at increased risk for poor MH outcomes. This generally tracks with risk for mental illness outside the pandemic experience [21, 49]. Given that gender-specific expectations and stressors have disproportionately affected females during the pandemic [4], it is clear why female gender was predictive in youth but less clear why it was not a determinant among adults in our study.

Non-binary individuals had greater risk for increased severity of psychiatric diagnoses in this study. Unfortunately, this group has been particularly avoidant of healthcare professional contact during the pandemic [58]. As such, MH care systems should optimally provide multiple options such as digital, telehealth and in-person sessions to accommodate individual preference and needs [37].

LGBTQ2S + status was a determinant of poor MH among Canadian youth and young adults. Potential explanations include exacerbated isolation in a previously socially isolated group [32], being quarantined with unsupportive parents [13] or in abusive environments [18] and increased peer victimization [22]. As such, targeted resources and support for Canadians in the LGBTQ + community are warranted. This may include provision of LGBTQ-affirming virtual extracurricular activities [25], social media MH resources such as the Trevor Project [18] and safe spaces [56]. It is advised to be particularly aware of intersectionally marginalized LGBTQ + groups such as those living in poverty who may not have access to digital technology [50].

#### Socioeconomic factors

Living in poverty increased risk for selected mental illness and for unmet support needs across youth and adult groups, doubling risks among adults over 30 years. Association between financial difficulties and poor MH was noted in Canadian first wave COVID-19 studies of children and adolescents [9, 47] and adults [44]. Exacerbation of negative impacts of poverty on MH have also been demonstrated globally [30], with amplification in those living with food insecurity [67]. However, to our knowledge, the link between poverty and failure to access MH support among Canadians during COVID-19 is novel. While this result may be unsurprising given pre-COVID-19 patterns, it is also particularly concerning given that Canada’s healthcare system aims to be universal. These results may reflect challenges in the system's capacity to either adapt to increased needs or maintain prior level of services in the context of the pandemic (particularly given inequities at baseline). Other identified socioeconomic determinants include limited educational attainment in adults, which relates to income potential, and ‘homemaker/caregiver’ occupational status among adults over 30 years old, which may limit financial autonomy and/or opportunities for social interaction. Regardless, it appears that COVID-19 exacerbated the role of poverty as a pre-existing disparity and action is needed to improve the availability and delivery of MH services to low-income populations.

#### Diagnosed conditions

Across all age groups, the lifetime diagnosis of a psychiatric illness was a determinant for current psychiatric problems and/or unmet MH support needs during the pandemic. This is generally consistent with the conceptualization that psychiatric illness can be exacerbated by stressful events [18]. Other conditions selectively conferred risk for current psychiatric problems and unmet MH support needs in either the youngest or oldest age group.

Children with special educational needs were significantly more vulnerable to negative outcomes. This may reflect the general vulnerability of these populations to stressful events or relate more specifically to the impacts of COVID-19 related changes (e.g, youth with special needs were less able to adapt to changes in school delivery). A Canadian study by Gonzalez et al. [23] found that while children with special educational needs received fewer clinical services during COVID-19, those who were also living in poverty, or who had parents with limited education or less than a full-time job were especially unlikely to receive services. As such, it is critical that these multiply disadvantaged families be targeted to receive tailored and individualized services to enable continuity of care throughout this and future pandemics. Moreover, support for their parents and caregivers is necessary to prevent burnout and their own MH challenges [1].

Medical illness was found to be a determinant of poor MH and unmet support needs in adults. These individuals were likely most directly impacted by delays or changes to health service provision as the system pivoted to prioritize COVID-19 patients. Indeed, McElroy-Heltzel et al. [35] found that among adults with illness, COVID-related loss of services was associated with mental distress, which was only partially buffered by the presence of social support. Maintenance of usual daily activities including exercise and social contacts is a recommended mitigation strategy to build psychological resilience and prevent mental illness onset among the medically ill during COVID-19 [31].

## Limitations

Despite recruitment efforts, our sample under-represented Canadians of Indigenous and East/Central Asian ethnicity, street-involved individuals and rural/northern residents, limiting the ability to examine these as possible determinants. Moreover, limitations inherent to online survey research including potential selection bias and volunteer effects may have had an impact on outcomes [12]. While increased severity of *any* psychiatric diagnosis was considered as an outcome, the presence of potentially new onset disorders other than depression, GAD and OCD were not systematically examined via diagnostic screens. In hindsight, for example, inclusion of an eating disorder screen would have been advisable, given emergent findings in youth [54].

However, the decision to include only three diagnostic screens was made in an effort to minimize participant burden and data validity impacts due to subject fatigue. In addition, diagnostic interviews were not conducted, although checklists may offer comparable results [7, 33]. Future research would benefit from the consideration of intersectionality (e.g, individuals with multiple determinants may be at disproportionately elevated risk for poor outcomes) while mixed-methods studies are also warranted to gain in-depth appreciation of individual narratives.

## Conclusion

Extending beyond the first wave, the COVID-19 pandemic has resulted in an ongoing and detrimental impact on the MH of Canadians, especially in young adults. Sadly, increasing disparities were observed in those already at risk for poor MH according to poverty/food insecurity, sex and gender minority status and prior psychiatric and health conditions. It is crucial that these findings be considered in optimizing interventions and policies to close the gap on pandemic-era and post-pandemic era MH inequities among Canadians.

## Supplementary Information

**Additional file 1: Table S1. **CHERRIES Checklist.

**Additional file 2: Figure S1. **Study Recruitment Relative to COVID-19 Waves in Canada.

**Additional file 3: Table S2.** Detailed characteristics of participants and Canadian reference samples.

**Additional file 4: Table S3. **Age-group specific rates of current GAD, depression, OCD as reported at baseline in PICS Sample and in Canadian (Cdn) pre-pandemic reference samples.

**Additional file 5: Document 1.** Study Protocol.

## Data Availability

The datasets used and/or analysed during the current study are available from the corresponding author on reasonable request.

## Abbreviations

GAD: Generalized anxiety disorder
CHERRIES: Checklist for reporting results of internet E-surveys
GAD-7: Generalized anxiety disorder scale
LGBTQ2S+: Lesbian/gay/Bisexual/transsexual/queer/2 spirit
MDD: Major depressive disorder
MH: Mental health
OCD: Obsessive–compulsive disorder
OCI-CV: Obsessive–compulsive inventory – revised (OCI-R) and -child version
OR: Odds ratio
PICS: Personal impacts of COVID-19 Survey
PHQ-9: Patient health questionnaire for depression
YA: Young adults
