# Supplementary material for: Age-specific determinants of psychiatric outcomes after the first COVID-19 wave: baseline findings from a Canadian online cohort study
Source: Child Adolesc Psychiatry Ment Health. 2023 Feb 6;17:20. doi: 10.1186/s13034-023-00560-8 (PMC9901839; doi:10.1186/s13034-023-00560-8)
Supplement: Supplementary file 1 — Additional file 1: Table S1. CHERRIES Checklist. [file 13034_2023_560_MOESM1_ESM.pdf]

Appendix Table 1: CHERRIES Checklist

	**Checklist for Reporting Results of Internet E-Surveys (CHERRIES)**	
**Item Category**	**Checklist Item**	**Explanation**
**Design**	Survey design	Online longitudinal survey using a convenience sample of anyone over the age of 8 years.
**IRB (Institutional Review Board) approval and informed consent process**	IRB approval	The study was approved by the University of British Columbia Children's and Women's Hospital Research Ethics Board
	Informed consent	This public survey was accessed online by first going through the informed consent form, which outlined the survey's investigators, purpose, duration, voluntary nature, confidentiality and privacy measures, and data storage process. This form included the study team's contact information that participants were encouraged to use if they had any questions and/or concerns.
	Data protection	The only identifiers collected were email addresses to send follow-up surveys to those who agreed to follow-up. This data was stored electronically on REDCap, which is password protected and only accessible to select delegates of the investigators.
**Development and pre-testing**	Development and testing	The survey was developed iteratively with stakeholders' feedback. The study team used REDCap to build the survey and tested it multiple times over a period of 3 weeks with multiple team members to time it and check for errors.
**Recruitment process and description of the sample having access to the questionnaire**	Open survey versus closed survey	This was an open survey available for anyone over the age of 8 years to participate in.
	Contact mode	There was no direct contact with each participant at the start but participants could reach out to the study team if they needed help. Participants reviewed the form and provided consent electronically on REDCap. From May to July 2021, participants were

		offered compensation at survey conclusion so they contacted the study team to request their gift card offered for survey completion. This compensation was removed because of a large influx of fraudulent data from records trying to claim the gift card.
	Advertising the survey	Social media ads were targeted where we looked at our sample profile to match it as closely as possible to the Canadian population. We reached out to special organizations to increase participation from certain groups e.g. specific cultural groups. Please refer to the appendix for the list of organizations contacted.
**Survey administration**	Web/E-mail	Participants were invited via a link to the e-survey sent through email. The survey was hosted on Research Electronic Data Capture (REDCap), a web-based software platform.
	Context	The study team had created the following study website to advertise the study: https://www.bcchr.ca/POP/our-research/pics This website was mostly visited by individuals looking to participate in a survey about COVID. All our media advertisements also directed interested participants to this website, where they could enter the study by first accessing the information and consent/assent forms.
	Mandatory/voluntary	The survey was voluntary so not every visitor to the website had to complete the survey.
	Incentives	Participants were entered into monthly draws where 10 participants were selected at random to win a $50 gift card. In addition, we also introduced a $10 gift for anyone completing the survey between May and July of 2021. This gift card, however, was removed as we noticed a large number of fraudulent records trying to claim this gift card.
	Time/Date	Baseline recruitment occurred from October 2020 to July 2021. Follow up surveys were also collected at each participant's 1 month, 3 month and 12 month post baseline survey completion.
	Randomization of items or questionnaires	We did not randomize items.
	Adaptive questioning	We did use adaptive questioning to reduce the number and complexity of questions.

	Number of Items	We had four types of survey so please see below for the item number for each:<br>Parent self-report: 6 cut to 4<br>Adult self-report: 6 cut to 4<br>Youth self-report: 6 cut to 4<br>Parent on child proxy: 7 cut to 5
	Number of screens (pages)	We had four types of survey so please see below for the approximate item numbers for each. The first number indicates the first version of the surveys which we later cut down to the second number:<br>Parent self-report: 14<br>Adult self-report: 14<br>Youth self-report: 10<br>Parent on child proxy: 11
	Completeness check	We did not do completeness checks before the survey was completed. All items had a non-response option.
	Review step	For the large majority of the survey, there was an option for participants to go back on a page and review their responses.
**Response rates**	Unique site visitor	We did not have access to IP addresses or cookies so we did not calculate view or participation rates.
	View rate (Ratio of unique survey visitors/unique site visitors)	We did not have a way of capturing this information.
	Participation rate (Ratio of unique visitors who agreed to participate/unique first survey page visitors)	The recruitment rate is 74.5%
	Completion rate (Ratio of users who finished the survey/users who agreed to participate)	The completion rate is 69.9%
**Preventing multiple entries from the same individual**	Cookies used	Cookies were not used; however, duplicate and fraudulent entries were avoided using the set of guidelines provided below under ‘Log file analysis.’
	IP check	IP addresses were not analyzed as it was not permissible by our research ethics board.

	Log file analysis	The following guidelines were used to identify fraudulent and duplicate responses:<br>1. Child/youth date of birth provided in parent self-report does not match the child/youth date of birth provided in the proxy surveys.<br>2. Email content including formatting being identical for different email accounts<br>3. Email addresses with random letters and lot of numbers.<br>4. Selecting 'yes' for child being vaccinated if child is under the age of 12 as children that young are not eligible to be vaccinated (at the time of survey administration)<br>5. Family configuration: selecting 'one caregiver household' on parent self report then selecting 'me and their other parent' for who takes care of the child in their home. Also selecting more than 1 as the number of homes the child lives in on the proxy survey<br>6. Selecting 'yes' for person of colour, after selecting 'Caucasian' for previous response<br>7. Selecting 'no' for person of colour, after selecting other ethnicities such as 'Black' or 'Latin American'<br>8. Completed the survey in an unreasonably fast time (<1 second per item on average)<br>9. Selecting "IOCDF" and "Anxiety Canada" as recruitment source for participants completing survey on or after May 18, 2021. Sudden influx of participants starting at this date, when active recruitment had occurred between November 2020 and March 2021.
	Registration	Our survey was an open survey.
**Analysis**	Handling of incomplete questionnaires	We only analyzed questionnaires from participants who completed at least the first section of the survey. However, it was not necessary to complete all sections of the survey.
	Questionnaires submitted with an atypical timestamp	We excluded questionnaires for which the participant completed a given section in a time that equates to less than 1 second per item on average.
	Statistical correction	No adjustment methods of this kind were used.
