# Supplementary figures and images for: Age-specific determinants of psychiatric outcomes after the first COVID-19 wave: baseline findings from a Canadian online cohort study
Source: Child Adolesc Psychiatry Ment Health. 2023 Feb 6;17:20. doi: 10.1186/s13034-023-00560-8 (PMC9901839; doi:10.1186/s13034-023-00560-8)

**Appendix Figure 1:**  
**Study Recruitment Relative to COVID-19 Waves in Canada**

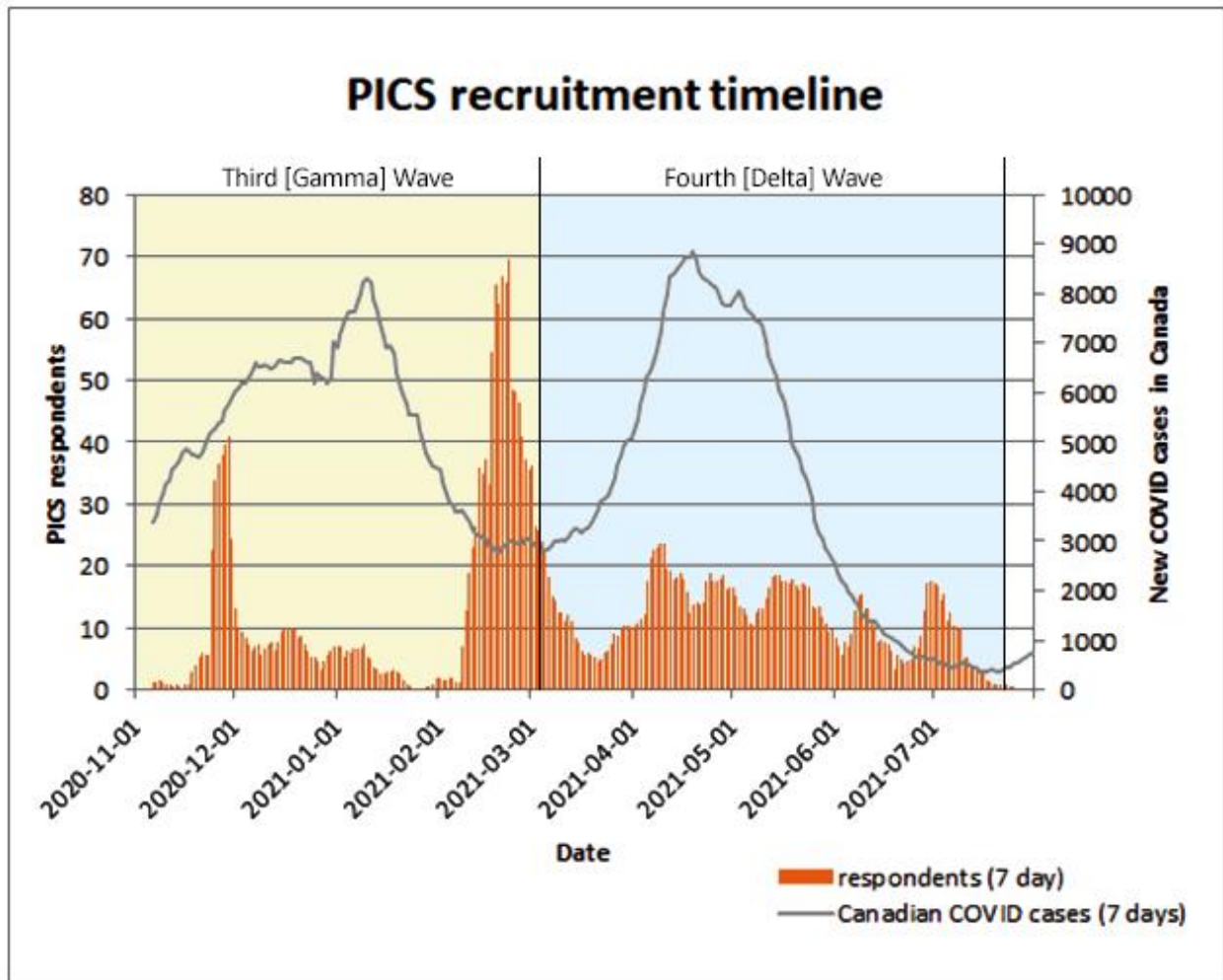

Supplement: Supplementary file 2 — Additional file 2: Figure S1. Study Recruitment Relative to COVID-19 Waves in Canada. [file 13034_2023_560_MOESM2_ESM.pdf]
