# Supplementary material for: Age-specific determinants of psychiatric outcomes after the first COVID-19 wave: baseline findings from a Canadian online cohort study
Source: Child Adolesc Psychiatry Ment Health. 2023 Feb 6;17:20. doi: 10.1186/s13034-023-00560-8 (PMC9901839; doi:10.1186/s13034-023-00560-8)
Supplement: Supplementary file 3 — Additional file 3: Table S2. Detailed characteristics of participants and Canadian reference samples. [file 13034_2023_560_MOESM3_ESM.pdf]

Appendix Table 2:
Detailed characteristics of participants and Canadian reference samples

	Survey detail	Canadian population reference[1]
**Gender**	female/male/non-binary	female/male
**LGBTQ2S+**	Lesbian/ Gay/ Bisexual/ Transsexual/Queer/ 2 Spirit	Lesbian/Gay/Bisexual (2015-2018)[2] 15-24 y = 6.4%; 25-64 y = 3.1%
**White**		indicating not being a visible minority.
**Immigrant**		<15 y=8.3%[3] ; adults=21.9%[4]
**Parent of 0-18 y**		37.5%[5]
**Child with special needs**		<15 y=10.9%; 15-19 y=4.6%[6]
**Rural setting**		16.1%[7]
**Northern setting**[8]	StatCan defined province-specific latitude cutoffs	
**Education** Less than bachelor degree	Among adults	Among 25-64 y
**Annual household income** < $75 000[14]		Among 15+ y
**Food Insecure**	"Very" or "Extremely" worried about family not having enough money for food in past 2 weeks	Among 15+ y[9]
**Number in home, mean (SD)**	2.48 (1.47)	2.4
**Lifetime psychiatric diagnosis**[10]	1064 (34%)	33.1%
**Alcohol/ substance use problem**		15-25 y= 12%; 45-65 y= 2%[11]
**Medical condition**	1268 (40%) As confirmed by a health professional: disorder of the heart, kidneys, immune disorder, diabetes/high blood sugar, cancer, arthritis, frequent/very bad headaches, epilepsy/seizures, serious stomach/bowel problems, and serious acne/skin problems.	0-18 y= 22%[12]; >19 y=44%[13]

Notes. Numbers (and percentages) are presented unless noted otherwise.
[1] Source unless otherwise noted: Statistics Canada. 2017.
[2] %Lesbian/Gay/Bisexual Canadians (2015-2018) 15-24 y = 6.4%; 25-64 y = 3.1%
[3] % immigrants <15 y https://www12.statcan.gc.ca/census-recensement/2016/as-sa/98-200-x/2016015/98-200-x2016015-eng.cfm
[4] https://www150.statcan.gc.ca/n1/daily-quotidien/171025/dq171025b-eng.htm?indid=14428-1&indgeo=0
[5]https://www12.statcan.gc.ca/census-recensement/2016/dp-pd/dt-td/Rp-eng.cfm?TABID=2&Lang=E&APATH=3&DETAIL=0&DIM=0&FL=A&FREE=0&GC=0&GID=1235625&GK=0&GRP=1&PID=109647&PRID=10&PTYPE=109445&S=0&SHOWALL=0&SUB=0&Temporal=2016&THEME=117&VID=0&VNAMEE=&VNAMEF=&D1=0&D2=0&D3=0&D4=0&D5=0&D6=0
[6]Human Resources and Skills Development Canada. (2011).
[7] https://www150.statcan.gc.ca/t1/tbl1/en/tv.action?pid=1710013501
[8] https://www150.statcan.gc.ca/n1/pub/21-006-x/2007007/6000446-eng.htm

[9] Polsky et al. 2020
[10] Includes psychiatric and substance disorders; Pearson, Janz and Ali. (2013)
[11] Pearson, Janz and Ali, 2013
[12] Queenan JA et al. 2021
[13] Public Health Agency of Canada, 2019
[14] Cost et al. 2022
