# Supplementary material for: Age-specific determinants of psychiatric outcomes after the first COVID-19 wave: baseline findings from a Canadian online cohort study
Source: Child Adolesc Psychiatry Ment Health. 2023 Feb 6;17:20. doi: 10.1186/s13034-023-00560-8 (PMC9901839; doi:10.1186/s13034-023-00560-8)
Supplement: Supplementary file 4 — Additional file 4: Table S3. Age-group specific rates of current GAD, depression, OCD as reported at baseline in PICS Sample and in Canadian (Cdn) pre-pandemic reference samples. [file 13034_2023_560_MOESM4_ESM.pdf]

Appendix Table 3:

Age-group specific rates of current GAD, depression, OCD as reported at baseline in PICS Sample and in Canadian (Cdn) pre-pandemic reference samples

**PICS Participants**	**Child <8 y (parent-report) N = 192**	**Youth 8-18 y (parent-report) N = 289**	**Youth 8-18 y (self-report) N = 137**	**Young adult 19-29 y (self-report) N = 586**	**Adult 30+ y (self-report) N = 1,936**
GAD	10 (5.2%)	69 (24%)	42 (31%)	319 (55%)	684 (38%)
Depression	8 (4.2%)	76 (26%)	48 (35%)	325 (56%)	839 (46%)
OCD	9 (4.7%)	52 (18%)	44 (33%)	136 (24%)	210 (12%)
**Ontario Child Health Survey[1]**	**Child 4 to 11 y (parent-report)**	**Youth 12 to 17 y (parent-report)**	**Youth 12 to 17 y (self-report)**	n/a	n/a
GAD	3.4%	5.5%	9.7%	n/a	n/a
Depression	1.1%	5.2%	7.3%	n/a	n/a
**Statistics Canada[2]**	n/a	n/a	n/a	**Young adult 15-24 y**	**Adult 25-64 y**
GAD	n/a	n/a	n/a	2.4%	3.0%
Depression	n/a	n/a	n/a	7.1%	5.0%

[1] Pre-pandemic Canadian reference 6-month rates (Georgiadis et al., 2019)

[2] Pre-pandemic Canadian reference 12-month rates (Pearson et al. 2013).
