# Supplementary material for: Age-specific determinants of psychiatric outcomes after the first COVID-19 wave: baseline findings from a Canadian online cohort study
Source: Child Adolesc Psychiatry Ment Health. 2023 Feb 6;17:20. doi: 10.1186/s13034-023-00560-8 (PMC9901839; doi:10.1186/s13034-023-00560-8)
Supplement: Supplementary file 5 — Additional file 5: Document 1. Study Protocol. [file 13034_2023_560_MOESM5_ESM.pdf]

Appendix Document 1 Study Protocol

Personal Impacts of COVID-19/Coronavirus Study (PICS)

Short Title: COVID-19 Personal Impact Study

PI: S. Evelyn Stewart
Co-PI: Hasina Samji

Background
The coronavirus disease 2019 (COVID-19) pandemic is an unprecedented global event within the 21st century. It is likely that its impacts will be widespread, extending beyond physical health aspects to include financial, social, familial and emotional sequelae for children, adolescents, their parents and other adults.

Public messaging has been dramatically mixed across time and global regions with respect to the potential danger of coronavirus and the best ways to prevent its spread. Individual responses to this pandemic may also vary widely, with personal influences extending beyond local authority messaging and local outbreak severity to include age, family factors, illness, health and occupation-related risk status, exposure history and available resources. This survey aims to capture longitudinal person-centred outcomes personal with respect to feelings, thoughts and actions, in addition to learning resources and coping techniques over a one-year period.

Aims and Hypotheses
AIM 1:
To characterize pandemic-era anxiety/mental health sequelae and their determinants.

AIM 2:
To determine pandemic-era mental health support needs and obtained resources.

AIM 3:
To identify pandemic-era uptake of online anxiety management tools, including perceived accessibility, utility, deficits and impacts.

Relevance:
While public health officials monitor infection and mortality rates of coronavirus/COVID, the short-term and long-term secondary effects on individuals' thoughts, feelings and actions may be overlooked. Identification of most effective resources and coping strategies is important for future individual and population-level interventions. This survey aims to capture impacts, their

correlates and predictors in a time-sensitive fashion, both during and subsequent to the pandemic (presuming resolution within a year).

Study Design

This study is a longitudinal survey to assess multidomain personal impacts of COVID-19 on child, adolescent and adult populations. An invitation to complete the survey will be sent via email or post mail, and following consent, a link will be unlocked to provide access to survey items. The survey responses will be deidentified and participation will be entirely voluntary.

At survey completion, level of interest or willingness to repeat the survey will be asked, in addition to optimal duration between surveys. Those agreeing to repeat the survey with linkage to their past survey data will provide an email (or postal address if preferred) for future contact.

Study Protocol

Potential participants will be sent an informational e-mail or mailed letter which describes details about the survey that are required for informed consent. Should participants have any questions, the e-mail addresses are provided for the PI and study staff for contact. We will aim to respond to all related emails or phone messages in less than one working day.

Upon reading about the survey, and following receipt of answers to their questions, interested potential participants will be invited to click a link that will direct them to an electronic consenting (e-consenting) module. For participants under the age of 19 at the time of enrolment, the electronic consent form will require the completion by both the participant (providing assent) and a legal representative (e.g. parent or guardian)(providing consent).

Participants at or over the age of 19 may sign consent for themselves and will not require parental/guardian involvement. Parent participants can choose whether their child, or children, if eligible, to participate in this study. Even if the child does not participate, the parent can still provide responses about their child/children (i.e. parent proxy on child). Those who will turn 19 years old by the time of study completion will be asked their contact information for us to recontact them for provision of consent after their birthday. Contact information, such as their e-mail address, will be collected during the assenting/consenting process to link their assent/consent response to their survey to minimize identifiable information on the survey responses. Participants providing assent are also asked to provide their e-mail to maintain privacy from parental involvement. Youth who do not have an e-mail address will be asked to provide the e-mail of their participating parent/guardian.

The consenting process for this survey will be assisted with a procedure approved by the legal department at PHSA and developed by individuals including the PHSA research privacy officer.

An information letter (contained within text of an email or in a mailed letter, as applicable) will provide an introduction to the survey study. This will state, 'Your electronic consent form will be stored in the BC Children's Hospital Research Institute's secured network in Vancouver, BC. Only authorized personnel will be able to access it.' After providing their full name and month and year of birth, completion of a field for a typed signature shall indicate consent (or assent in the case of children and youth) to participate in the study. This will specify that one has read and understood the information about the study; that they voluntarily consent to participate in this study; that they have had the opportunity to ask questions and obtain satisfactory responses; that they understand their responses will be deidentified; that if they choose to enter a personal email address for future contact, this will be stored separately from their survey response; and that they can refuse to participate or to withdraw from the study at any time.

Following completion of consent and assent, participants will be connected to one of two surveys: one for the parent proxy and adult self-report version, and one for the child/youth self-report. Survey completion is expected to take between 15-30 minutes for each respondent with respect to themselves and each child (where applicable). No item responses will be required to proceed to the next survey item.

At the conclusion of the survey, participants will be asked their opinion as to whether the survey should be sent again as a follow-up. Individuals who participate in follow-up surveys will have their current answers linked with future survey answers, but not their identity or identifying information. It will be made clear that their email address and consent forms will be stored separately from their survey responses at the BCCHR.

For those providing an email address and willingness to conduct a follow-up, emails will be sent with links to the survey thrice four times in total; thrice at one-month, 3-month and 12-month following completion of the initial survey, and once in March of 2022 to capture data about the fifth wave of the pandemic from all respondents at one timepoint regardless of when they completed the baseline survey. Of important note, this frequency will be changed if it is found to diverge notably from overall participant feedback- and an ethics amendment will be submitted prior to changing frequency of follow-up surveying. Respondents are made aware that they can participate in any follow-up surveys.

Given that children, youth and adults may be struggling emotionally or with mental health issues at this time, efforts have been made to direct individuals to freely available community resources at two points. This information will be provided in the introduction letters for consent and assent (for those who choose not to participate but who desire support) and will be presented again following survey completion (in the event that answering survey questions has caused distress).

Recruitment

Participants will be invited via email or by written posted letter (when email address is unavailable), to complete the survey from source types, as follows:

1) The BCCH Patient Experience Office will email their list of approximately 85 families who have previously expressed interest in completing surveys or questionnaires. This has been confirmed by Aidan Scott of the BCCH Patient Experience Office.

2) The BCCH Provincial OCD Program has a list of previous research participants (comprising OCD-affected children, their siblings and healthy controls) who have expressed willingness to be contacted in the future for additional studies. Specifically, these research participants are recruited from OCD Registry (H11-01759) and OCD Neurocognition (H12-02626). These individuals will be invited for participation.

3) Researchers who hold email contact lists for children, youth or adults previously giving permission for future contact about research studies will send informational emails and letters to these individuals. The REB numbers of the studies where invitations will be sent from include: H16-01794, H18-00671, H18-03183, H14-01900, H17-03353, H18-00671, H19-00293.

4) Through a process as informed by Holly Longstaff at the PHSA, permission will be requested to obtain names and mailing addresses (or emails if available) of active BCCH patients from Patient Medical Records (PMR). Of note, no highly sensitive information will be requested. An approved ethics protocol and accompanying protocol will be required to make that request. Given the expected time lag (estimated 51-90 days for approval and data access based upon PHSA website), it is possible that contacted individuals may have previously been contacted about or completed this survey through another means. As such, an additional item will be added to the introduction letter, in which this is asked. Those identifying themselves as previous/current survey study participants will be excluded to prevent duplication. Prospective participants invited through PMR will be sent an invitation letter (physical mail or email) on behalf of Dr. Jana Davidson, Chief Medical Officer at BCCH. These letters will be sent by research staff at the Provincial OCD Program acting as a representative of PHSA. All research staff conducting this has completed all requirements to do so (e.g. PHSA Client Contract Agreement), with the process developed and approved by Holly Longstaff.

5) Letters, posters, and flyers with a web address of the study description will be placed within the BCCH hospital, within the community, on social media (e.g. Facebook parent groups, Twitter with relevant organizations, such as BC Family Services Institute), and on professional, not-for-profit organization websites (such as AnxietyCanada and the International OCD Foundation) to invite a diverse array of participants. Study members would circulate the poster to professional networks (e.g. Responsive Intersectoral Children's Health, Education, and Research (RICHER) Initiative at BC Children's Hospital) to be disseminated in communities through e-mail by other network members. Interested individuals will be able to access the study invitation, and following consent/assent will be directed to the survey. AnxietyCanada will be sending out the invitation letter to their subscribers.

6) Social media influencers and other influencers with a large following of audiences will be sent information about the study that includes a web address with the study information.

7) Organizations that cater to underrepresented groups such as certain ethnic/racial communities or low SES groups will also be contacted to increase engagement from these communities.

Inclusion Criteria
All individuals who provide consent (or assent with consent by a parent or guardian) will be included for study.

Exclusion Criteria
Individuals who do not consent (or for whom consent is not provided by their legal guardian) will be excluded. Those who do not understand written English will be ineligible for study.

Measures
The survey battery has been restricted to an estimated 20-30 minutes for completion for children, youth and adults, in efforts to minimize participant burden, and optimize likelihood of follow-up survey completion. Participants answering questions about the youth in their care will take an estimated additional 20 minutes. Data for children and youth will be captured from a combination of child/youth self-report (8-18 y) and parent proxy reports (for those with children 0-18 y); parents will also be asked to complete self-report measures (for those with children of all ages). Should adults without children wish to participate, they will only complete adult self-report measures.

Battery measures comprise: 1) the 'Personal Impacts of Coronavirus/COVID Survey (PICS)' and 2) a standardized screen for obsessive-compulsive disorder (and follow-up questions if screening positive.

1) The 'Personal Impacts of Coronavirus/COVID Survey (PICS)' was developed by the PI and Co-investigators with iterative versions informed by feedback from parents, children, youth, child and adolescent psychiatrists and psychologists and researchers. Developmentally appropriate language has been used for child/youth versus adult versions of the PICS survey. These have also been piloted on children, youth and adults to identify completion time and comprehensibility.

2) Mental illness screens and measures
Depression:
Patient Health Questionnaire and
Patient Health Questionnaire modified for adolescents

Generalized Anxiety Disorder:
Generalized Anxiety Disorder 7-item (GAD-7) scale (Spitzer et al., 2006)

Obsessive-Compulsive Disorder Screen and Severity Measures:
Child self-report, parent report and adult self-report versions of the Obsessive Compulsive Inventory (OCI): Revised (OCI-R 12) and Child Version (OCI-CV) and the child and adult versions of the Yale-Brown Obsessive Compulsive Scale (Y-BOCS) and Children's Y-BOCS (CY-BOCS) will be used.
3) The Needs and Supports questions and the MyAnxietyPlans (MAPs) questions, completed by parent proxy and adult self-report, was developed by the PI and Co-investigators with iterative versions informed by feedback from parents and adults. It seeks to better understand the extent supporting resources were obtained since the pandemic and the perceived accessibility, utility, deficits and impacts of an online anxiety management tool from AnxietyCanada.
Reimbursement
All participants will be enrolled in a draw for a gift card, with each family member that participates regarded as a separate entry in the draw. Every month, a maximum of 10 gift cards valued at $50 each will be randomly drawn, with one gift card for each entry. Should there be a month with 10 or fewer entries, then the number of gift cards for that month would correspond to the number of entries (i.e. 5 entries for a specific month means 5 gift cards).

Data Storage
Personal identifiers will not be used on any of the data forms or samples; a code-number will be assigned to each participant's data. Code numbers are 3- or 4-digit numbers based on chronological order of recruitment. The masterlist of the participants and their codes will be held by Dr. Stewart and select research team members, and kept in a password-protected file on a password-protected computer that is in a locked office. Only Dr. Stewart and the research team directly involved with this study will have access to the data and samples at processing and at analysis. A current list of the study personnel and their duties will be kept on file and updated as needed. The password on the computer will be changed on a regular basis.

Data Security
The BCCHRI Clinical Research Support Unit (CRSU) stores REDCap survey data in a secure fire-wall-protected server in Vancouver. There is a web application server that is the only gate to connect to the Database server, where the de-identified information is stored. Following the Freedom of Information and Privacy Protection Act of BC and The Personal Information Protection and Electronic Documents Act of Canada is paramount to the BCCHRI CRSU. Unlike standard data management systems in which a study database contains a collection of electronic case report forms, REDCap Survey creates surveys that are independent of each other. The main role within the REDCap system that controls the set-up of these surveys is the Super Administrator. As a "Super Admin", not only can surveys be created but they can also be copied and used as a template for another survey. At the BCCHRI, only a designated person from the Clinical Research Support Unit is a Super Administrator. The Super Admin is also the role

within the system that establishes accounts and user access for other types of roles per survey. When creating new accounts, the Super Admin can turn on or off specific, standard data management user account features to enable/disable data management roles such as the Administrator, Designer, collector and monitor of survey data, and receiver of completed survey notifications. None of the roles that the Super Admin creates allow for data that was previously entered in the survey to be changed however, "read-only" and data extraction rights can be granted. User accounts are only set up for research data management, the backend, personnel and they are all electronic signatures comprised of a username and password. All accounts created require the legal first and last name of the account holder, and their approved institutional email address. New accounts are set up with a temporary password assigned by the Super Admin and it is required to be changed by the account holder upon first-time sign-in to REDCap Survey. Only the account holder knows the newly assigned password but the Super Admin or Administrator can reset it at any time. User accounts can be reused for other surveys as directed by the investigator or delegate.

No individual results with identifying data will be reported back to the participants or to anyone else. There are theoretical non-physical risks associated with taking part in this study. For example, disclosure of sensitive data could result in discrimination by employers or insurance providers, although the chance that de-identified research data would be released is estimated to be small. Research records and medical records identifying subjects may be inspected in the presence of Dr. Stewart or her designate by representatives of the UBC Research Ethics Board for the purpose of monitoring the research. No information or records that disclose subjects' identities will be published without their consent, nor will any information or records that disclose their identity be removed or released without consent unless required by law. The master list that matches participant names to their unique identifier that is used on all research-related information will not be removed or released without consent unless required by law. There are no clear foreseeable circumstances where disclosure of identifying data may be required by law.

Given that we expect that some children, youth and parents will be struggling emotionally and with mental illness, a list of community resources, including the BCCH Kelty Mental Health Resource Centre, HeretoHelp.bc.ca and the suicide hotline, will be noted prior to consent/assent and again at completion of the survey. Participants will be encouraged to reach out to these resources if they are struggling. We do not require identifying or contact information for survey participation. As such, it would not be possible to provide intervention based upon individual item responses. This limitation is clarified prior to survey assent and consent.

Statistical Analysis

Primary analyses will investigate time-dependent trends in the measures of mental health, quality of life, cognitive functioning, and family functioning using a mixed-model regression framework

with both fixed and random intercept and time effects. This will allow for the characterization of the average within-child and within-parent changes in the measures of interest, as well as quantification of variation across children and parents. Non-linear time effects will be included to account for the possibility different rates of change in mental health and family functioning over the course of the study period. This analytic framework will be applied to the evaluation of cognitive, socioemotional, behavioural and physical status factors (Aim 1) and to characterizing resources, coping strategies, perceived effectiveness in dealing with the coronavirus pandemic (Aim 2), as well as the perceived accessibility, utility, deficits, and impacts of online anxiety management tools (Aim 3).
